# Fluid-Structure Interaction in Abdominal Aortic Aneurysm: Effect of Modeling Techniques

**DOI:** 10.1155/2017/7023078

**Published:** 2017-02-22

**Authors:** Shengmao Lin, Xinwei Han, Yonghua Bi, Siyeong Ju, Linxia Gu

**Affiliations:** ^1^School of Civil Engineering and Architecture, Xiamen University of Technology, Xiamen, China; ^2^Department of Mechanical and Materials Engineering, University of Nebraska-Lincoln, Lincoln, NE 68588-0656, USA; ^3^Department of Interventional Radiology, The First Affiliated Hospital, Zhengzhou University, Henan Province, China; ^4^Nebraska Center for Materials and Nanoscience, Lincoln, NE 68588-0656, USA

## Abstract

In this work, the impact of modeling techniques on predicting the mechanical behaviors of abdominal aortic aneurysm (AAA) is systematically investigated. The fluid-structure interaction (FSI) model for simultaneously capturing the transient interaction between blood flow dynamics and wall mechanics was compared with its simplified techniques, that is, computational fluid dynamics (CFD) or computational solid stress (CSS) model. Results demonstrated that CFD exhibited relatively smaller vortexes and tends to overestimate the fluid wall shear stress, compared to FSI. On the contrary, the minimal differences in wall stresses and deformation were observed between FSI and CSS models. Furthermore, it was found that the accuracy of CSS prediction depends on the applied pressure profile for the aneurysm sac. A large pressure drop across AAA usually led to the underestimation of wall stresses and thus the AAA rupture. Moreover, the assumed isotropic AAA wall properties, compared to the anisotropic one, will aggravate the difference between the simplified models with the FSI approach. The present work demonstrated the importance of modeling techniques on predicting the blood flow dynamics and wall mechanics of the AAA, which could guide the selection of appropriate modeling technique for significant clinical implications.

## 1. Introduction

Abdominal aortic aneurysm (AAA), the local dilation of infrarenal aorta, is related to the weakening of arterial wall. When AAA grows bigger, the rupture occurs with a reported mortality rate of 90% [1]. The maximum diameter of the AAA, that is, 55 mm, and its growth rate of 10 mm/year are generally used for making surgical decisions to prevent its rupture [2]. However, clinical studies also reported a rupture rate of 12.8% [3] or 23% [4] for AAA less than 55 mm in diameter. It is clear that a better rupture predictor is needed for the prevention of AAA rupture.

From the perspective of mechanics, the rupture of the AAA happens as the blood induced wall stress exceeds the arterial wall strength. Arterial wall stress could better predict the rupture potential of the AAA than the geometry information only. In addition, blood flow induced shear stress was recognized for contributing to the degradation of elastin and the red blood cell damage, which in turn elevates the wall stress and accelerates the AAA growth [5]. Computational modeling is generally used to estimate these mechanical predictors and the resulting rupture potential of AAA.

Considering the pulsatile blood flow and nonuniform pressure distribution inside the AAA sac, a fluid-structure interaction (FSI) model is desired to capture both the blood flow dynamics and the deformation of AAA wall [6]. However, this technique is significantly time consuming and usually simplified as either a computational fluid dynamics (CFD) model or a computational solid stress (CSS) model. The CFD approach treats the aneurysm wall as rigid to quantify the blood flow dynamics in terms of shear stresses and vortex formation [7, 8]. The CSS model replaces fluid domain by a time-varied pressure to characterize the AAA behavior in terms of wall stresses and compliances [9]. Both simplified modeling techniques have been extensively used in the study of AAA. With the advancement of computing capacity, the impact of the aforementioned simplifications on wall stresses is attracting more attentions. Scotti et al. [10] have quantified the difference between the FSI and CSS model; the later treats the blood flow as a uniform distributed pressure load. They observed that the CSS underestimates the peak wall stress by 10.2% and 30.2% in cases of uniform wall thickness of 1.5 mm and variable wall thickness from 0.5 mm to 1.5 mm, respectively. The modeling techniques induced large variations on wall stresses did not hold valid in the study by Leung et al. [11]. They stated that modeling techniques resulted in minimal variations in terms of wall stresses distribution and peak stress magnitude on three patient specific AAAs. Despite this controversy, only one study [12] was found by comparing the flow dynamics within the aneurysm using either FSI or CFD modeling technique. It was observed that CFD, compared to FSI, led to overestimation of flow shear stresses.

Motivated by the controversial opinions on the efficiency of CSS on AAA as well as limited comparisons between FSI and CFD, the main objective of this work is to systematically study the impact of simplified modeling techniques on the AAA behavior. The role of material constitutive model of AAA wall was also investigated. Results will provide a better understanding of the interactions between blood flow and AAA wall.

## 2. Materials and Methods

### 2.1. Geometry and Mesh

**Figure 1:**
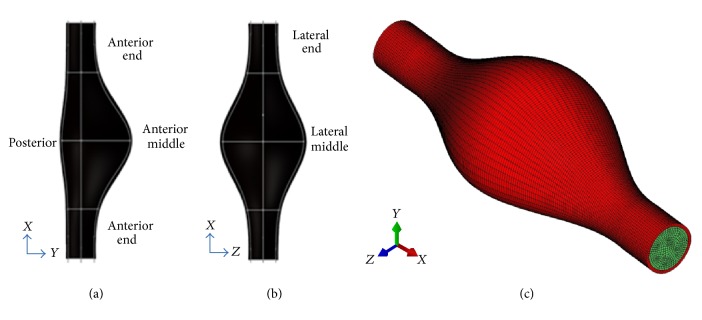
AAA profile in the (a) sagittal plane and (b) coronal plane; (c) finite element model with integrated fluid (green) and solid (red) domains.

A generalized AAA geometry, recommended by surgeons [13, 14], is adopted and recreated using commercial software SolidWorks (Dassault Systèmes SolidWorks Corp., Concord, MA) as shown in Figures 1(a), 1(b), and 1(c). The asymmetric AAA shape was bounded by two edges in the sagittal plane and two symmetric ones in the coronal plane. These edges are defined by the five curved as specified in Table 1. The positions of these edges are defined by five different curves, which are represented by the following exponential equation:

(1) $$\begin{array}{c} y\;\;or\;\;z\;\;coordinate=C_{0}+{\left(\;\frac{C_{1}}{\sqrt{\pi C_{3}}}\;\right)}^{P_{2}}{\left(\;e^{-\left(x^{2}/c_{2}\right)}\;\right)}^{P_{1}}, \end{array}$$

where *x* is the axial position varying between 0 and 190 mm. Four coefficients *C*_*i*_ (*i* = 0  to  3) along with two exponents *P*_1_ and *P*_2_ are associated with each curve, as listed in Table 1.

The created AAA has a maximum inner diameter of 64.8 mm along the lateral direction and 54.6 mm along the anterior-posterior direction, respectively. Both the proximal neck and distal neck are 21.6 mm in diameter. The arterial wall is assumed with a uniform thickness of 1.5 mm. The total length of the model is 190 mm. The mesh convergence study in terms of fluid pressure and wall displacement was performed. The AAA wall was meshed with 13398 C3D20RH elements using Hypermesh (Altair Engineering, Tokyo, Japan) while its lumen was meshed with 79982 HEX8 using Gambit (ANSYS-Fluent Inc., Lebanon, USA) as shown in Figure 1(c).

### 2.2. Constitutive Model

The anisotropic material model of AAA wall was adopted from the work by Holzapfel et al. [16], which is defined by the strain energy density function as follows:

(2) Wwall, anisoC=C10I−1−3+k12k2ek2I−4−12−1.0+k32k4ek4I−6−12−1.0,

where *W* is the strain energy per unit of reference volume; $\overset{-}{C}=F^{T}F$ denotes the right Cauchy-Green tensor where **F** is the deformation gradient; ${\overset{-}{I}}_{1}=tr\bar{C}$ is the first deviatoric strain invariant and ${\overset{-}{I}}_{4}=n_{0}\cdot \overset{-}{C}\cdot n_{0},{\overset{-}{I}}_{6}=m_{0}\cdot \overset{-}{C}\cdot m_{0}$ are the pseudo-invariants of $\overset{-}{C}$ where **n**_0_ and **m**_0_ are direction vectors for two families of collagenous fibers arranged in a double-helix pattern. The material parameters for this constitutive model are adopted from study by Raghavan et al. [17] as *c*_10_ = 110 kPa, *k*_1_ = *k*_3_ = 210 kPa, and *k*_2_ = *k*_4_ = 1700. The orientation angle *θ*  (cos⁡*θ* = **m**_0_ · **e**_*θ*_ = **n**_0_ · **e**_*θ*_) between each fiber families and the circumferential direction of the wall is 43°.

To investigate the role of simplified material models, a softer isotropic hyperplastic model of the AAA was defined by a polynomial strain energy density function as

(3) $$\begin{array}{c} W=C_{10}\left(\;\bar{I_{1}}-3\;\right)+C_{20}{\left(\;\bar{I_{1}}-3\;\right)}^{2}, \end{array}$$

where two material constants were adopted as *C*_10_ = 0.174 MPa and *C*_20_ = 1.881 MPa [18].

### 2.3. FSI Modeling

**Figure 2:**
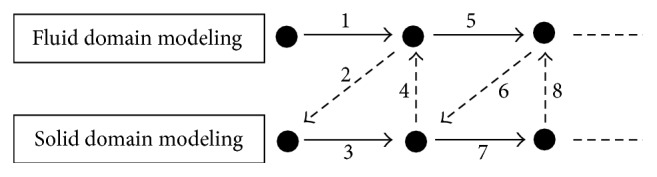
FSI iterative coupling illustration.

The FSI model was developed through a commercial software Mpcci 4.2 (Fraunhofer SCAI, Germany). The iterative coupling scheme between the solid and fluid domains was illustrated in Figure 2. Specifically, the calculated pressures from fluid domain modeling were passed to the solid domain serving as external loadings. The deformations calculated from solid domain modeling were then passed to fluid domain adjusting its Eulerian boundary. The number in the diagram shows the sequence of algorithm execution. Number 1 represents the first modeling step in the fluid domain modeling, that is, CFD, to obtain the pressure field. The pressure passing process was designated as Number 2. The solid domain modeling was then used to calculate boundary deformations, represented by Number 3. The boundary deformations were passed back to CFD referred to as Number 4. The process was iterated until convergence. The time steps for fluid model and solid one are 1 ms and 5 ms, respectively. The coupling is implemented at every 5 ms. The passing parameters between fluid domain and solid domain are the pressure and deformation at each integration point of the element.

The solid domain analysis was carried out through ABAQUS 6.12 (Dassault Systèmes Simulia Corp., Providence, RI, USA). Both ends of wall are constrained in all degrees of freedom [10, 19]. The deformation of AAA wall altered the volume of fluid domain, which was handled by inducing a moving coordinate system using the Arbitrary Lagrangian Eulerian method [20]. Two dynamic meshing techniques, including smoothing and remeshing, were used to maintain the mesh quality during the movement of the fluid domain.

The blood flow dynamics was governed by the momentum and mass conservation equations as below.

(4) Mass:  ρfΔ·v=0Momentum:  ρf∂v∂t+ρfv−df•·∇v=∇·τf+ffB,

where *ρ*_*f*_ is the fluid density, **τ**_*f*_ is the fluid stress tensor, **f**_*f*_^*B*^ are the body forces per unit volume, **v** is the fluid velocity vector, $\overset{•}{d_{f}}$ is the moving coordinate velocity, and $v-\overset{•}{d_{f}}$ is the relative velocity of the fluid with respect to the moving coordinate velocity.

**Figure 3:**
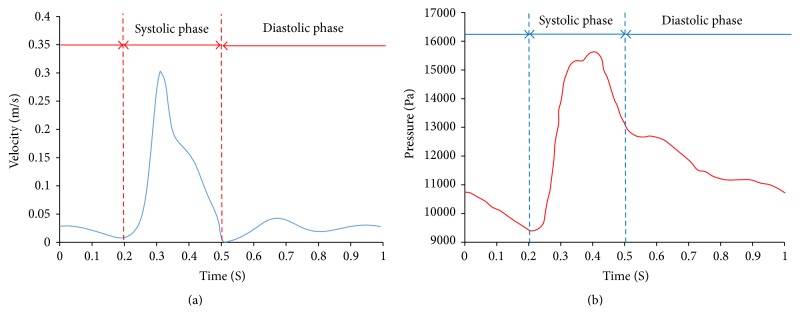
(a) Inlet pulsatile velocity with peak value at *t* = 0.3 s and (b) outlet pressure with the peak value at *t* = 0.4 s [15].

These governing equations were solved using the commercial software FLUENT (ANSYS® Academic Research 13.0). The blood was assumed as a Newtonian fluid with a density of 1035 kg/m^3^ and a dynamic viscosity 0.0035 Pa·s based on a large shear rate of approximately 300 s^−1^ in aorta [21]. The pulsatile inlet velocity and outlet pressure for one cardiac cycle, shown in Figures 3(a) and 3(b), were adopted from the literature [15]. The turbulence intensity of a fully developed flow was estimated as

(5) $$\begin{array}{c} I=0.16Re_{d_{h}}^{-1/8}, \end{array}$$

where the Reynolds number, Re_*d*_*h*__, was calculated as 191 at the inlet, which corresponds to a turbulence intensity of 8.3%. The characteristic length *d*_*h*_ is defined as the inlet diameter. The* k*-*ε* model was used to accommodate the possible nonlaminar effect. Total of three consecutive cardiac cycles were simulated to ensure the flow stability.

### 2.4. CSS Modeling

The CSS modeling technique simplifies the FSI model by treating the fluid domain as an uniform distributed pressure load with the same profile as the outlet pressure (Figure 3(b)). It only provides information on wall mechanics.

### 2.5. CFD Modeling

The CSF modeling technique focuses on the fluid domain only and assumes that the AAA wall is rigid. The numerical parameters for all three modeling techniques are consistent.

## 3. Results

The FSI modeling of AAA simultaneously captured the blood flow dynamics and wall stress field. In contrast, CFD modeling could only provide information on the fluid dynamics, and CSS modeling only provides the wall stress field. These simplified modeling techniques are compared with the baseline FSI results.

### 3.1. Effect of Arterial Compliance on the Blood Flow Dynamics: FSI versus CFD

The blood flow dynamics within the AAA is generally simulated using either CFD or FSI technique. The major difference between these two techniques is considering the arterial compliance or not. The role of arterial compliance on the prediction of fluid dynamics is then investigated by comparing results from CFD and FSI methods.

**Figure 4:**
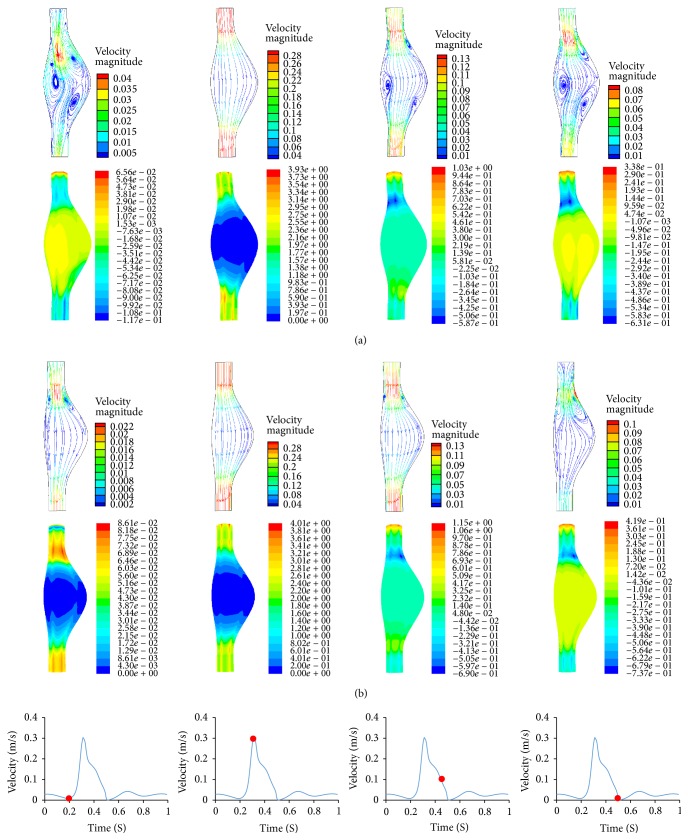
Flow streamlines colored by velocity magnitude (m/s), wall shear stress (Pa) at four different time points (from left to right: 0.2 s, 0.3 s, 0.45 s, and 0.5 s). (a) FSI model and (b) CFD model.

Figure 4(a) depicted the FSI calculated flow velocity distribution on the sagittal plane and wall shear stress distribution of aneurysm wall at four time points of one cardia cycle. During the acceleration phase (0.2 s to 0.3 s), the flow remains laminar and fully attached to the bulging walls. This is due to the fact that the temporal acceleration of the flow is larger than the convective deceleration caused by the widening of the aorta. This results in a positive pressure gradient along the axial direction. It is clear that the flow velocity reduced toward the aneurysm bulge. High magnitude (about 4 Pa) of wall shear stress is found at the two normal end sections and a large gradient is observed as the aneurysm expands. At the bulge region, the magnitude of wall shear stress is dropped to a very low magnitude.

Dynamic vortices are reinitiated and developed during the deceleration part of the systolic cycle from 0.3 s to 0.5 s. The flow deceleration induced the adverse pressure gradient which initiated four vortices inside the aneurysm (two at the proximal neck, one at posterior side of bulge region, and one at anterior side of distal neck). Large negative wall shear stress (maximum −0.587 Pa) is found at the proximal neck region which corresponds to the region of vortices.

During diastolic cycle, that is, from 0.5 s to 0.2 s of the following cardiac cycle, the flow velocity remains at low magnitude and the pressure continues to drop. It is interesting to note that a small pressure gradient alternates between positive (450 Pa) and negative values (−140 Pa), corresponding to the flow acceleration and deceleration at small magnitude. The vortices continue to grow in size and detached from proximal neck.

**Figure 5:**
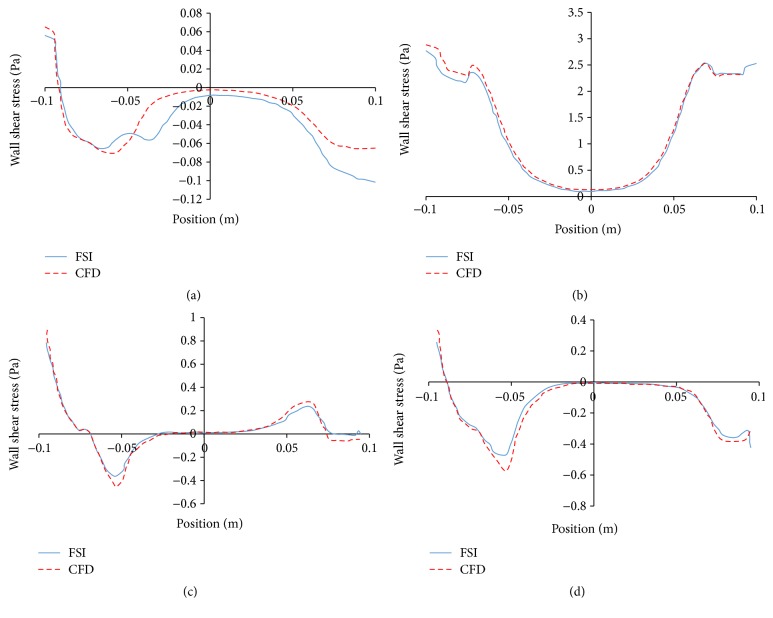
Average wall shear stress along axial direction at four different time points from FSI modeling and CFD modeling: (a) 0.2 s, (b) 0.3 s, (c) 0.45 s, and (d) 0.5 s.

The CFD-predicted flow dynamics is illustrated in Figure 4(b). Regardless of the modeling simplification as a rigid wall, flow dynamics shows similar trend, that is, the vortex initiated and developed during flow deceleration and diminished during flow acceleration. However, two vortices stayed at the proximal neck unlike the detachment of vortex in FSI model. This led to relatively larger shear stress at two ends and smaller one at bulge region (Figure 5). The major difference among these two models in predicting the wall shear stress is at the proximal neck region; the CFD model overestimates the wall shear stress by 30% compared to the FSI model at the deceleration phase. This difference is corresponding to the increased vortex intensity at the proximal neck in CFD model.

### 3.2. Wall Mechanics of AAA

Most of numerical models on predicting arterial mechanics adopted the CSS modeling technique. The impact of blood flow was simplified as the uniform distributed pressure loading. To evaluate the efficiency of CSS on the fracture prediction of AAA, the wall mechanics of AAA from both FSI and CSS was compared.

**Figure 6:**
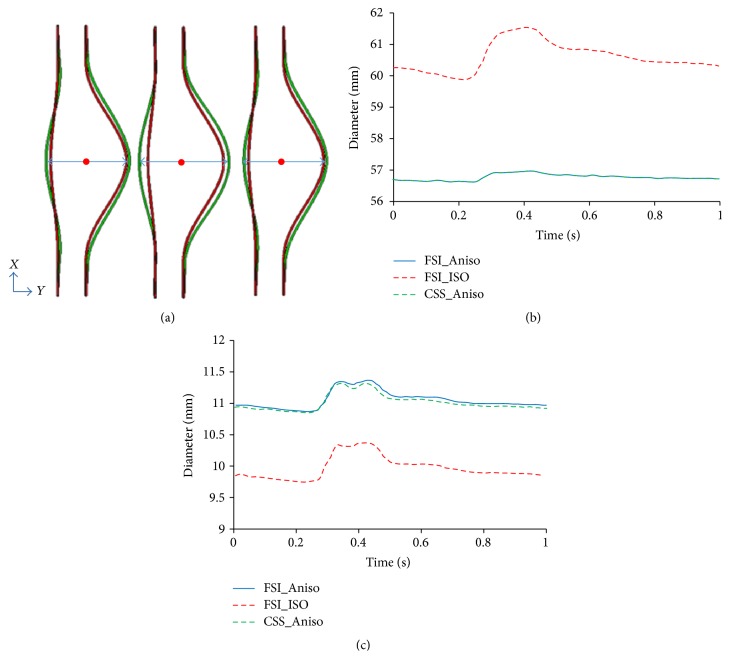
(a) Wall motion at peak systolic pressure for FSI model with anisotropic wall properties (FSI_Aniso), FSI model with isotropic wall properties (FSI_ISO), and CSS model with anisotropic wall properties (CSS_Aniso). From left to right: FSI_Aniso, FSI_ISO, and CSS_Aniso; (b) maximum diameter of AAA time history; (c) center point at the maximum diameter of AAA time history.

**Figure 7:**
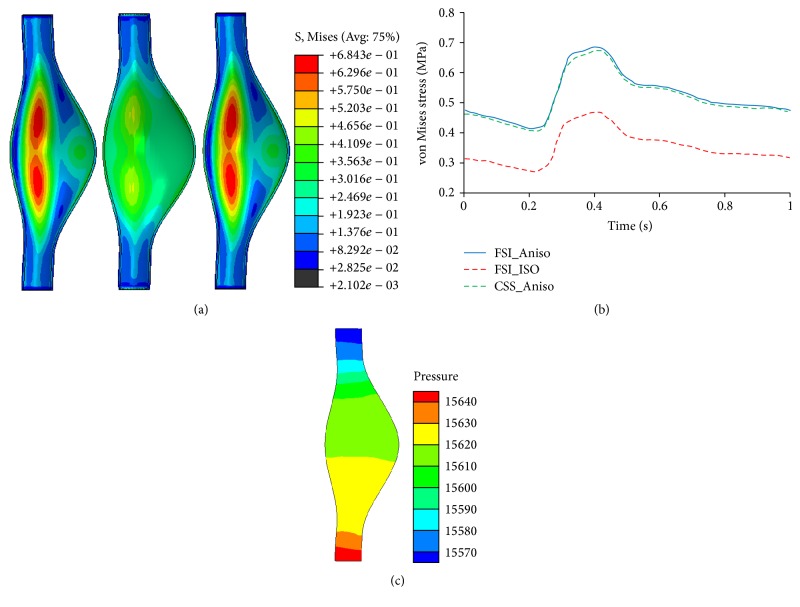
(a) Contour of von Mises stress (MPa) for three models (from left to right: FSI_Aniso, FSI_ISO, and CSS_Aniso) at peak systolic pressure; (b) time history of peak von Mises stress (MPa); (c) pressure (Pa) distribution at peak systolic pressure in FSI mode stress.

Figure 6(a) demonstrated the wall deformation induced by FSI model and CSS model at peak systolic pressure. The red line indicates the original AAA shape while the green line indicates its current shape. Similar wall motions were found from both FSI and CSS models considering anisotropic wall properties. The FSI obtained maximum diameter of AAA in the sagittal plane overlaps with the one obtained from CSS model as shown in Figure 6(b). A slightly shifting motion toward anterior side is observed from the CSS model in comparison to the FSI model as shown in Figure 6(c), which depicted the center point of the AAA maximum diameter. In terms of wall stresses, no distinguishable difference is found in terms of both von Mises stress distribution and peak von Mises stress history (Figures 7(a) and 7(b)). This was attributed to the minimal pressure drop across AAA sac. At peak systolic pressure, only 70 Pa (less than 1%) pressure drop is found from the inlet to outlet as shown in Figure 7(c).

### 3.3. Material Properties of Aneurysm Wall Influence the Choice of Modeling Technique

An isotropic aneurysm wall was also considered in FSI models (Figure 6) in comparison with the aforementioned anisotropic wall models. The larger wall motion as well as smaller wall stresses were observed with the softer isotropic AAA wall. Specifically, the maximum diameter of AAA at the peak systolic pressure is 61.5 mm in the isotropic FSI model, while it is 57.0 mm in the anisotropic model. The center shifting toward the anterior side is smaller than that in anisotropic model. In addition, the maximum von Mises stresses on the aneurysm wall are 0.467 MPa and 0.673 MPa for the isotropic and anisotropic model, respectively.

**Figure 8:**
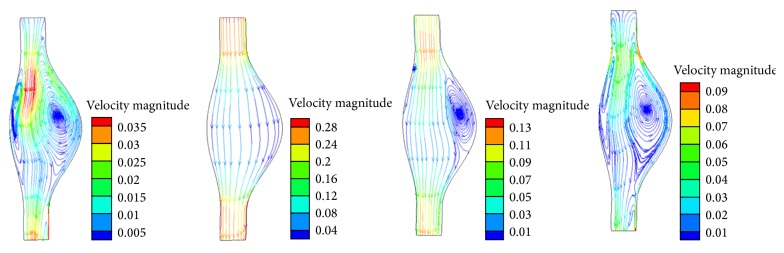
Flow streamlines colored by velocity magnitude (m/s) at four different time points (0.2 s, 0.3 s, 0.45 s, and 0.5 s) for FSI model with isotropic wall property.

The blood flow behavior is also affected by the material property of aneurysm wall. The flow dynamics in the FSI isotropic model, shown in Figure 8, is compared with the results form FSI anisotropic model (Figure 4(a)). The common features are that vortex is generated at the flow deceleration and remains stable in the diastolic phase. However, unlike the vortex structure in anisotropic model (Figure 4(a)), the bulge area deformed more toward the anterior side than the posterior side. This results in unbalanced size of vortex in this sagittal plane.

## 4. Discussion

Three major modeling techniques, that is, FSI, CFD, and CSS, were symmetrically investigated for better understanding the wall mechanics and blood flow dynamics of the AAA. The coupling between wall compliance and flow dynamics of the AAA was fully captured using FSI approach. Dynamic lumen pressure and wall deformation at each integration point of the element are exchanged every 5 ms. The biaxial tests of human AAA tissue specimens conducted by Rodríguez et al. [22] were adopted as the anisotropic material model of the AAA wall. The simulated flow pattern was verified by our mesh convergence study, as well as the published experimental observations [14]. The pressure difference from the inlet to outlet alternates between positive and negative values, corresponding to the flow acceleration and deceleration (Figure 4(a)). The flow deceleration during each cardiac cycle induced the initiation and progression of four vortices, which correspond to increased wall shear stress. Our observations are consistent with other numerical work [12, 23] in terms of life cycles of vortex. The observed shifting motion toward anterior side (Figure 6(c)) is due to the fiber orientation in the anisotropic material model. This is consistent with the study by Rissland et al. [24] who also considered the anisotropic nature of AAA wall. In addition, the AAA was extensively described as isotropic material models [18]. If the AAA wall property is altered from the anisotropic model to a softer isotropic one, the maximum AAA diameter in the sagittal plane becomes larger due to the reduced stiffness in the circumferential direction. However, the shifting motion toward anterior side is minimal in the isotropic model. The increased AAA diameter might aggravate the flow disturbance and thus the difference between FSI and CFD modeling techniques. Moreover, significant reduced wall stress is found in the isotropic model, which agrees with the observations by Rodríguez et al. [25] and Geest et al. [26]. Although the wall shear stress is generally several orders of magnitude lower than the wall stress, its effect on wall inflammation, thrombus formation, calcification, and breakdown of the wall integrity is not trivial [27].

The CFD model technique without considering the wall compliance significantly affected the flow dynamics within the AAA as illustrated in Figure 4. Rigid wall assumption in CFD model resulted in relatively smaller vortices. This was due to the fact that rigid wall impedes the energy dissipation into the aneurysm wall, which in turn increased the turbulent kinetic energy at the vortex region and overestimated wall shear stress at the proximal neck region. Our observations are in agreement with the experimental work by Deplano et al. [14] and numerical work by Khanafer et al. [12]. The implications using a simplified CFD model include underestimation of disrupted endothelial cell alignment as well as the associated tissue remodeling [28, 29].

The wall mechanics of the AAA and its associated rupture potential are usually obtained using a CSS approach, which simplified the blood flow dynamics by a time-varying pressure profile. For a relative stiffer anisotropic wall, minimal differences in stress distribution and wall deformation were found between FSI and CSS approaches. This is due to the minor pressure drop inside the aneurysm, which equalized the boundary conditions between FSI and CSS modeling approaches. The relatively constant pressure field inside the AAA has been demonstrated in other studies [6, 30] and negligible wall stress differences between the CSS and FSI techniques were also reported in the work by Leung et al. [11]. However, the controversy comes from the work by Scotti et al. [10], who stated that the CSS technique underestimates the AAA wall stress by 10.2% or larger considering the wall with varying thickness. This discrepancy is caused by the axial length of the AAA. Specifically, Scotti et al. adopted a longer AAA length of 240 mm instead of 190 mm in our work. In comparison, Leung et al. [11] used a much shorted patient specific AAA with a length ranging from 96 mm to 134 mm. The increased AAA length, especially the neck length, will result in larger resistance of the flow and thus a larger pressure drop. This implied that CSS model might underestimate the wall stresses, compared to FSI model, unless the pressure profile close to the aneurysm could be used in the CSS model. This way, the CSS model is reasonable to predict the AAA rupture risk.

In the present model, a generalized asymmetric AAA geometry is adopted [13, 14]. Although more and more studies tend to use the patient specific morphology reconstructed from medical images for predicting the rupture potential of AAA [11, 24, 31], a recent study by Reeps et al. [32] demonstrated that modeling techniques have more influence on the AAA characterizations than patient specific morphologies. We examined the influence of two existing material models of aneurysm wall on the outcomes of FSI modeling. Considering the natural variation of wall material properties, a sensitivity analysis of material coefficients on the outcomes of modeling techniques might provide further information regarding how the difference between modeling techniques could vary in response to varied wall material properties. The AAA wall was assumed as homogeneous materials without considering the three-layer wall structure and the variations between the neck and sac regions. The constraint from the surrounding tissue was also neglected in this work. Despite these simplifications, the impact of modeling techniques on the AAA behavior was justified considering the comparative nature of this work.

In summary, this study systematically investigated the role of modeling techniques and material models on the AAA behaviors. FSI approach is preferred for presurgical planning of the AAA. CFD predicted that flow dynamics within AAA might underestimate the development of vortices and overestimate the shear stress. A softer wall material will aggravate the differences between these two modeling approaches. In addition, the CSS model might underestimate the wall stresses unless the pressure profile within the aneurysm could be adopted. The present work demonstrates the differences of three popular modeling techniques on predicting the AAA behaviors, which can be used to provide a fundamental understanding of the AAA behavior, especially blood flow dynamics and wall mechanics, to guide the selection of appropriate modeling technique for preclinical planning and to illuminate the possibilities for exploiting their potential to prevent AAA rupture.

## Figures and Tables

**Table 1:** Coefficients to define curves of AAA model shapes [13, 14].

Curve	*C*_0_ (mm)	*C*_1_ (mm)	*C*_2_ (mm^2^)	*C*_3_ (mm^2^)	*P*_1_	*P*_2_
Lateral near end	10.2	100	7	0.8	0.009	0.85
Lateral middle	10	100	10	0.8	0.007	0.75
Anterior near ends	9.7	100	8	0.8	0.0095	0.95
Anterior middle	10.5	100	9	0.8	0.006	0.81
Posterior	10.4	100	8	0.8	0.008	0.39
